# Pyorrhea and Prophylaxis

**Published:** 1917-09

**Authors:** F. H. Skinner


### PYORRHEA AND PROPHYLAXIS

BY F. H. SKINNER, D.D.S.

Pyorrhea work is the hardest we have to do in dentistry. It requires patience, delicate manipulation, a knowledge of what we are doing and the confidence and cooperation of the patient. In order to secure the confidence and cooperation of patients, we must educate them to appreciate what we are doing sufficiently to induce them to give all the home care necessary to complete the work done by the dentist.

### ETIOLOGY

Pyorrhea is caused primarily by infectious substances which locate on the tooth surfaces. Fermenting .soft accumulations infect the gums and cause inflammation. There is always a serous fluid oozing from an inflamed surface. Serumal deposits, socalled, are the precipitate from this serous exudate. This deposit may form at the apex of a root as the result of an apical abscess, on the side as a result of a alveolar abscess or just under the free margin of the gum from ginvivitis, and work rootwise. There are a great many predisposing conditions which lessen the resistance of the tissues, thereby making it possible for local infection to take place. Following are some of the most prominent:

Malocclusion, which causes bruising of the peridental membrane, is probably one of the principal predisposing causes. In the mouth of the average person who has reached thirty-five or forty years of age, some portions of the teeth have worn more rapidly than others, and the portions thus left high produce a side pressure, causing injury of the peridental membrane and absorption of the process or else forcing the teeth apart at the contact points, which allows fibrous foods to impinge the crest of the gum septum. In other words, such teeth do not occlude anatomically. Poor contact points probably rank second as a causative factor; next a lowering of the general vital resistance; then a great many minor causes, such as imperfectly formed enamel surfaces, enamel surfaces which have become etched from deposits left there until acid fermentation has taken place, fillings improperly finished at the gingival margins or fillings which can not be made smooth, such as all of the phosphate and some of the silicate cements. All banded crowns and regulating appliances cause some irritation and a great deal, if carelessly fitted. Bands which fit, but which have been driven up until they impinge the peridental membrane, are also a great source of irritation.

Pyorrhea is found also, almost without exception, where one tooth has been lost. For instance, when the first permanent molar has been extracted, there is a tendency for for space to become closed and for the opposing teeth to elongate. The teeth posterior to the space come forward as a result of the pulling on the fibers due to the formation of scar tissue where the tooth was extracted. In a short time, malocclusion produces a force which causes the remaining teeth to tilt still more, and in a few months, most of the stress of mastication is brought to bear on the under side of the tilting tooth, where the tissue has become bruised. Here also is usually found an accumulation which is a source of infection to the already injured tissues, and pyorrhea results. Unless the full space is maintained by the proper interlocking of the cusps of the remaining teeth, I believe it is perfectly legitimate to cut into the tooth at each end of the space and insert a bridge, preferably of the spur and inlay type. This method of making a short bridge maintains the space and occlusion, and yet allows normal movement and function of the teeth in the alveolus without the danger of loosening the inlays. I do not believe in removing pulps when it can possibly be avoided, but should it be necessary, the tissues surrounding the teeth should be made healthy beforehand.

### THE TEETH AND THEIR SUPPORTING TISSUES

A tooth is composed of the dental pulp, dentin, cementum, which covers the root, and enamel, which covers the crown. The dentin is filled with canaliculi, which, in the crown, extend to the inner portion of the enamel, sometimes even permeating it, and in the root portion, they extend to the cementum, occasionally pass clear through it and form canals of greater or lesser size which connect with the peridental membrane. The outer surface of the cementum has a rough, pitted, irregular surface to which the peridental membrane and dental ligaments gain very firm attachment. The density of the cementum is greatest just under this rough surface. At the cemento-enamel junction, the cementum is very thin, but increases considerably in thickness as it approaches the apex of the root. Between the dentin and the cementum is the granular layer of Tomes. In the cementum of the apical third there are many spaces known as lacune. Although it can not be demonstrated with a microscope and is disputed by many, some authorities believe the lacune have direct connection with the dental tubuli, which, of course, would mean that they were directly connected with the dental pulp. Sealing in twenty-five per cent. ethereal pyrozone illustrates this.

The teeth occupy sockets in the alveolar process, which is a bony support formed around the teeth as they erupt. It is very porous, spongy, has thin edges and is somewhat of an insecure framework for supporting the great stress of mastication brought to bear upon it. The teeth are suspended in these sockets by dental ligaments, some of which run obliquely upward, forming a sort of hammock, and are attached to the process by fibers which spring from the cementum and appear in bundles. The cementoblasts build up the layers of cementum, and then calcify both the latter and the fibers, which, in this way, become attached very firmly to the root surfaces. A short distance from the surface of the root, they usually break up into smaller bundles which anastomose and interlace, passing around blood vessels and other fibers and again uniting into larger bundles for attachment at their other extremity. These bundles of fibers, or ligaments, gain very firm anchorage in the alveolar process, and some bundles gingivally to the crest, pass from the proximal side of one tooth to the proximal side of the adjacent tooth. Through the mucous membrane and vessels which pass through the alveolar process, the peridental membrane is supplied very plentifully with blood. The blood vessels pass over the crest of the process, as well as through it, and permeate the peridental membrane. Any inflammation at the crest of the gum usually causes some sclerosis, thus shutting off a part of the blood supply.

### SUSPECTED AVENUES OF INFECTION

There are certain glandular structures in the peridental membrane which have been beautifully described by both Black and Talbot. These glandular cells are in direct connection with channels leading into the mucous membrane and alveolar process. Any infectious material on the teeth or accumulating between the teeth, at the free margin of the gum, has an uninterrupted avenue of entrance into the deeper structures. Microorganisms, introduced into the tissues through pyorrhea pockets or blind abscesses, when not taken care of by leucocytes or antibodies, travel uninterruptedly to where they are taken up by the circulation and distributed to points of lessened resistance, where they form colonies, and produce different diseases, according to the organs infected.

The absorption of microorganisms and their circulation in the blood stream, into which they are introduced through the tissues surrounding the teeth, are, today, considered responsible for many diseases. The microorganisms are carried by the blood stream to the joints, heart, kidneys, or other organs, where they produce destructive changes, which, for a great many years, were unaccounted for. Research workers have compared the bacteria taken from tooth surfaces, their surrounding tissues and apical abscesses with those taken from remote parts of the body, and found them apparently identical. In fact, it is known that bacteria are selective in the tissues which they inhabit. Those taken from an apical abscess of a patient with a diseased heart and injected into lower animals, if they fertilize at all, usually select the heart tissues for their habitation. Cultures of bacteria, taken from blind, abscessed or pyorrhea pockets and injected into rabbits or guinea pigs, produced the same pathological conditions in the animal as those from which the patient was suffering. Many systemic infections have been cleared up by the treatment of ill-kept mouths, and the extraction of abscessed teeth, followed by curettement of their sockets.

The canals leading from the inner wall of the ginviva into the deeper structures are demonstrated readily by the use of the Dunlop vapor. When this vapor is properly applied, these channels can be traced through the soft tissues and alveolar process for a considerable distance. I have introduced the vapor in the region of a cuspid, had it travel through the alveolar process and gum tissues and come bubbling out around the neck of a third molar. I have introduced it at a bicuspid on the right side, had it cross the median line and come bubbling out around the bicuspids on the left side. I have introduced it between the buccal roots of an upper first molar and had it penetrate down into the tonsils or up into the forehead, showing that channels actually extend from the inner wall of the gingiva into deeper tissues, in almost any direction. following the line of inflamed tissue. In fact, according to Dr. Hartzell, the direct lymphatic drainage of the tissues surrounding the teeth is the most perfect of the whole body, affording a direct access to the deeper structures of the neck and mediastinum, thereby making it possible for microorganisms introduced into the tissues through a pyorrhea pocket to travel uninterruptedly into the deeper structures of the body.

### TREATMENT

Up to five years ago, the most successful treatment generally known consisted of the thorough removal of all deposits, peridental membrane and the rough outer surface of the cementum down to the hard layer, wherever the soft tissues had lost their attachment, followed with the best polishing possible, and then with maintained cleanliness. In seventy-five per cent. of the cases, the congestion would disappear and the swollen tissues recede to where the peridental membrane was attached to the teeth, if the work were thoroughly done.

Usually, by the slight proliferation of bone cells and the formation of scar tissue, the teeth were held very firmly for years. If prophylaxis were kept up, a reinfection of the tissues was not liable to occur. In nearly every case, it was possible to stop suppuration and produce apparent health in the tissues surrounding these teeth, but it was necessary to splint or anchor all loose teeth in order to maintain them with any degree of permanency. In extensive cases, some form of removable bridgework which forms a positive splint is absolutely necessary.

Since the introduction of the Dunlop vapor, my treatment of pyorrhea has differed somewhat; now I am combining this vapor with the use of the planing instruments.

A great many patients whose mouths show no inflammation at the gum margins have osteitis or osteomyelitis, which results in the disintegration or absorption of the alveolar process. Red or cyanotic areas high up on the gums indicate diseased conditions and lowered vitality, and in periods of cessation of oral prophylaxis, the result is infection, suppuration and pyorrhea. The Dunlop vapor, if understood and properly applied, will reach these congested areas. If recognized in time, the development of pyorrhea can be prevented by establishing oral prophylaxis, eliminating all irritation and building up Nature's resistance by the use of the paste and vapor.

In the treatment of advanced cases of pyorrhea, I first inject Dunlop Pyorrhea Paste into all pockets, then remove deposits and stains from every portion of the tooth not covered by the gums and polish all roughened, etched or undeveloped enamel surfaces. This sometimes requires several sittings. This scaling and polishing should be carried a millimeter or two under the free margins of the gums, but at this time, I do not attempt instrumentation in deep pockets. Crowns, bridges, carelessly finished fillings, unsavable teeth, or any other irritant, which may be present, must be corrected or removed. If any cusps are too long and cause lateral stress on any of the teeth when the jaws are brought together and the teeth gritted from side to side, and if the teeth can not be depressed into the sockets, the cusps should be ground so as to relieve all undue stress, because regeneration of tissue can not take place while there is malocclusion. Poor contact points also must be corrected. If fillings which lack contact points are present, I frequently drill a little cavity in the filling and pack that with white base plate gutta-percha. This gradually gains space between the teeth and also forces the other teeth together, thereby reestablishing practically normal contact points, and when sufficient space has been gained, well contoured fillings with good marginal ridges and fissure restoration should be inserted, and any cusps which interfere with proper occlusion should be ground. I especially speak of marginal ridges and fissure restoration, because so many fillings, instead of being built up with marginal ridges and fissures, are inserted with flat surfaces which slope toward the contact point, thus producing force which tends to separate the teeth. Nature normally provides a high ridge near the point of contact, which forces the food toward the occlusal fissures, and unless we follow out this thought in our repair work, there is sure to be trouble sooner or later.

After all debris has been removed from the teeth and the coronal surfaces thoroughly polished, including a millimeter or two just under the free margin of the gum, I spray the mouth with ethylborate and reinject all pockets with the paste, and use the pocket packer. The next day it will be noticed that a great deal of inflammation has disappeared, and the patient will say his mouth feels better. Then I begin using the Dunlop vapor treatment, which consists of introducing borated oxygen gas into the tissues by means of a very delicate, blunt needle. Penetration of the tissues by the vapor can be detected in three ways: 1st, by the flashes seen as it passes through the little channels; 2d, by a crackling sense of touch to the finger, and 3d, the sensation felt by the patient. Only the smallest amount that can be detected in any of these three ways should be given at the first treatment, which should be given around the second molars and the cuspids. The tissues should never be punctured with this needle. By passing it just under the free margin of the gum, and then bearing outward a little, the vapor will find openings in the soft tissues. These are little culdesacs, ducts or glands which Dr. Talbot describes in his ''Insterstitial Gingivitis.'' One will be surprised to see the distance the vapor will penetrate, whereever inflammation exists. The needle should never be carried to the bottom of deep pockets, nor should it be used against tissue in which new granulations are beginning to form, because if we once break up or blow out newly granulating tissue, fresh granulations will have great difficulty in reforming. It is advisable to give a treatment every day for five days in succession, then let the tissues rest for ten days or two weeks, when another series of five treatments should be given. The vapor should always be introduced into the more healthy areas and allowed to penetrate through them to the diseased portions.

Within three or four days, accumulations will begin to gather on the teeth. After the vapor treatment has been administered, paint the teeth with the disclosing solution, and after rinsing his mouth, let the patient see, by the use of a handmirror, how rapidly infectious accumulations, as evidenced by the stain, have gathered on his teeth. At this point, I begin to teach him how to take care of his mouth. In order to prevent reinfection, it is essential to prevent this debris from gathering on the tooth surfaces. The patient is provided with a kuroris, or cotton carrier of some sort. I prefer the kuroris, because it is especially designed for this purpose, and the handle end can be used as a tongue scraper. He is provided, also, with a polisher, dental tape, mouth mirror and a supply of cotton rolls, and many patients even wish to use the disclosing solution.

It requires preseverance to teach some patients to use this outfit, while others learn to handle it very rapidly, but a dentist must be able to use it in his own mouth before he can be successful in giving instructions in its use. I do not believe that we have the ability to induce our patients to do that which we are not able to carry out ourselves, and I always feel that the dentist who is giving advice to patients which he is not carrying out for himself, is not really sincere, and his arguments lack force, if he is not actually doing the work for himself.

Until the teeth are smooth, and while new tissues are forming, I do not advocate vigorous rubbing of the gums with the cotton rolls or brush, because, if the tooth surfaces are not smooth, rubbing the gums intensifies the irritation, and if new tissue is forming, we are liable to destroy it and thereby arrest the repair work; also, the formation of new bone cells around the teeth is restricted by the hastened contraction of the tissues around the necks of the teeth, as a result of hard rubbing, but the tooth surfaces, themselves, should be kept clean by the use of cotton rolls, cheese cloth napkins or wooden points. The proximal surfaces should be cleaned with tape; a reasonable amount of brushing immediately after each meal, with the use of the dental tape between the teeth will remove most of the loose debris which accumulates.

I want to go over my patients' teeth with a polisher once or twice a week or have them do so. This is essential to prevent reinfection during the healing process. The paste and vapor stimulate circulation and reduce inflammation, and the paraffin pocket packer protects the tissues while healing. Occasionally deposits seem to soften, or will detach themselves from the teeth with very little force. In these cases, I expect a true reattachment of the tissues to the peridental membrane. In other cases, they seem to produce little or no effect, except to reduce the inflammation and make the mouth feel more comfortable. After a couple of weeks of vapor treatment and cleanliness, if suppuration has not ceased, I begin surgical treatment of the roots, but in all cases at this stage of procedure, I want to remove the deposits only, trying not to injure the peridental membrane, for we are working for a reattachment of the gum tissues and their return to normal condition.

This vapor has an affinity for diseased tissues, and it is surprising to see how it will penetrate both the soft and bony. It can not be forced into healthy tissue. Red or cyanotic areas located on the gums, well away from the teeth, indicate osteitis or osteomyelitis and can always be reached by this treatment when properly applied. It establishes more comfort for the patient, produces healthy hyperemia and apparently stimulates circulation.

SURGICAL INSTRUMENTATION

In some cases the use of the Dunlop preparations apparently causes no change in the deposits or their attachment to the teeth. In these cases thorough instrumentation is indicated.

A good many of the socalled pyorrhea instruments on the market would make better gouges and chisels than dental instruments. A great many men fail in pyorrhea work on account of the selection of instruments which are not delicate enough to do the work and which are improperly shaped. More depends upon the man than on the instruments, but any man will do better work with properly designed instruments which he has learned to handle correctly than he will with certain classes of instruments which have been forced upon the profession for years. The planing instruments are in a class all by themselves. I would not recommend that any man purchase a set of these instruments without taking a course to learn how to handle them.

Next to the planing instruments, I would recommend the Younger set, but with these it is frequently necessary to use local anesthetics hypodermically, which usually cause soreness of the tissues, which may last for a few days or a few weeks. I do not believe the tissues ever recuperate as quickly after hypodermic injections as they do without them.

From the histology of a tooth, one can readily see that an instrument which removes a very thin, delicate shaving and leaves a smooth surface, such as the planing instruments, should be used, for they can not be made to cut deeply, and are adaptable to any shape of root surface, with the exception of the sharp angles where roots bifurcate, in which case an instrument of the Younger type is preferable. When the planing instruments have been used, soreness usually leaves the soft tissues quickly. In some cases, where tissues have been badly lacerated from the use of improper instruments, patients have been ill from anaphylaxis, or the hypersusceptibility to the action of toxins introduced into the tissues and absorbed. The planing instruments are shaped so that they extend to the bottom of pockets and yet cause very little laceration of the tissues.

All etched or roughened enamel surfaces adjacent to soft tissues must be made smooth and polished. Then all deposits should be removed, and the necrotic peridental membrane and the irregular outer surface of the cementum should be planed to the hard layer. This is absolutely essential where the soft tissues do not reunite to the peridental membrane and close the pocket, because millions of bacteria will propagate in the dead peridental membrane and other debris which adheres to the irregular, pitted surface of the cementum. Leucocytes form a very dense wall, which, in a large measure, protects the soft tissues from the infection of microorganisms. Leucocytes possess ameboid movement, but they are unable to cross over from the soft tissues to the infected root surfaces in sufficient numbers to cope with the millions of bacteria which grow on improperly cared for tooth surfaces. The leucocytes in the soft tissues adjacent to pyorrhea pockets gradually die, and in connection with broken down or degenerated tissue, are thrown off in the form of pus.

Therefore, it is absolutely essential, where thorough instrumentation proves necessary, that all root surfaces should be planed to the hard layer, but care should be taken not to go through this hard layer, for just underlying it in the middle third, we run into the granular layer of Tomes, or at the apical third, into the lacunae, either of which if opened, exposes hypersensitive surfaces and spaces where millions of bacteria will again propagate. With proper instruments it is very easy to tell when this hard surface is reached. To quote from Dr. Hartzell: "In planing off lime salt deposits, the instrument feels as if it were scraping over unpolished marble. In planing off the necrotic periosteum, the sensation is that of pulling the instrument over silk velvet. A few strokes will remove this, and we then come to a hard but rough surface. With a few strokes of a sharp instrument over this surface, the sensation is that of rubbing a burnisher over polished ivory. This is the time to stop, for we have reached the hard layer which lies just under the rough surface of the cementum. If we are unfortunate enough to plane through this layer, the instrument will begin to jump. This indicates that we have cut too deeply and have done as much damage as we have done good, for these areas are large enough to harbor millions of pus-producing bacteria.''

If the patient should be negligent about his mouth and deposits should get started again after the work has been thoroughly done, instruments which do not cut should be used on the surfaces which already have been planed, for we do not want to take any chance of cutting through the hard layer. The little files known as ''Smith Trimmers'' or those designed by Drs. Hartzell, Tompkins, Hutchinson, Towner, and many others, are probably the best adapted for secondary treatment. They soon lose their edge, thus eliminating the danger of cutting too deeply, yet they will remove deposits from a smooth, hard surface.

Many cases of pyorrhea can be cured by thorough instrumentation. The Dunlop preparations, properly applied, greatly increase the percentage, and, I believe, where it has been used as heretofore described, and where some of the fibers of the peridental membrane still retain their vitality, the attachment of tissue will take place much nearer the neck of the tooth and less instrumentation will be required to obtain good results. Cases which do not respond to these treatments are usually complicated with some systemic disease, which should be diagnosed and corrected. Diabetes, Bright's disease, intestinal toxemia, pernicious anemia, and, in fact, any of the slow, chronic, debilitating diseases so reduce the general vitality that tissues do not respond as readily as they should. Often, in cases of this kind, oral conditions will respond immediately upon correcting the systemic disorders, but very often, the systemic disorders are corrected when the oral cavity is put in a hygienic condition and cleanliness maintained.

Vaccines have proved of value in some cases, but I believe when a vaccine is used, it should be autogenous.

There is a form of suppuration which is frequently mistaken for pyorrhea and for which one should be on his guard, *i. e.*, where an apical abscess has opened along the side of a root and is discharging at the neck of the tooth. Sometimes these will respond to vapor treatment, but frequently it is necessary to open the gum, excise the apex of the root, and curette all necrotic and carious bone. Plane the root thoroughly, as you would in a pericemental abscess, then pack with gauze and euroform paste and make the opening heal from in the bottom. We do not always get the results we hope for from this treatment, but it usually stops the destruction of bone and will make the tooth useful for a number of years.

Patients should be informed that pyorrhea is the result of bacterial growths which have been allowed to form and remain on the tooth surfaces. They should also be told that their teeth will require constant care in order to maintain a healthy condition after the pyorrhea has been cured, and that this cure can be maintained permanently only with their cooperation, because the same cause will produce the same result again.

While the toothbrush, in a large measure, is a failure in preventing decay and pyorrhea, I am not quite ready to eliminate it as a toilet article, but I am very particular as to its shape and its manner of use. Too gritty a powder, which will wear away the teeth, especially where the gums have receded, should not be used. Never allow patients to prick their gums with the bristles of a brush, for toothbrush bristles are never sterile, and injured places on the gums form points of entrance for infecting organisms, and later will result in scar tissue, which contracts, thereby starting recession of the gums. Small brushes are preferable to large ones. Also have patients practice brushing their teeth before you, until they learn to handle a brush correctly. You will find that they have acquired the habit of starting to brush in one place, usually at the gingival margins of some of the anterior teeth. This can be detected in its early stages by a slight recession of the gums above the teeth where the brush is first placed. Later on, this can not escape our notice, because at this point the gums have receded and grooves have been cut in the teeth. Usually patients will tell you this is erosion, but this kind of erosion stops when they desist from using gritty tooth powders and from starting to brush at the same point.

The chief benefits derived for the use of a toothbrush are that it acts as a series of fine tooth picks, thereby removing a part of the loose debris, and that the massaging produces a more healthy condition of the gums, but massaging the gums can be carried to excess. I do not like to see the gums present a hard, leathery, mottled, light pink surface, for this calloused appearance is the result of severe rubbing. There is not good circulation, and infection, followed by serumal deposits, usually occurs sooner or later, and works rootwise under this kind of tissue. Gum tissue should lie over well formed bony structure and should have a good, bright, healthy color and a network of fine capillaries showing plainly just under the surface. I do not mean hyperemia, but just a good healthy circulation. As far as I have been able to observe, daily massaging with cotton rolls or cheese cloth napkins increases circulation, but does not cause the gums to become calloused. It is better to take toothbrushes away from patients, if they can not learn to handle them properly, and have them depend upon the polisher, dental floss and cotton rolls, for with these they will not injure their teeth or gums.

With the aid of a looking glass and a mouth mirror, a patient can see foreign substance on his own teeth after applying the disclosing solution. Polishing with wooden points will remove all soft accumulations located by the stain. When necessary, patients are allowed to use an abrasive such as Pepsodent tooth paste, precipitated chalk or a fine silex preparation. I have not used pumice stone in my practice for many years, because it does not polish highly enough to inhibit the formation of bacterial plaques.

Some patients are able to maintain clean tooth surfaces for six months or even longer, while others who will not try or who have not the ability, need to have a prophylaxis treatment every thirty days. We must find out how long they are able to keep their mouths in a clean, healthy condition, and then establish that length of time as a period for prophylaxis treatments. In this work it is necessary to notify patients when they are due, and when informed of the plan used for keeping in touch with them, they invariably wish to be put on the call-up list.

The results obtained by those doing prophylaxis work may not coincide in every way with scientific theories, but are obtained clinically, and clinical results are convincing and are what we are after.

Dr. Loeb in his book, "The Mechanistic Conception of Life," says: "Life of warm-blooded animals—man included—ends with cessation of oxidation in the body. As soon as oxidations have ceased for some time, the surface films of the cells, if they contain enough water and the temperature is high, become permeable for bacteria, and the body is destroyed by microorganisms."

We can not think of a more favorable place for the breaking down of tissue cells than is found in the alveolar process and gum tissues, where there is moisture, body temperature, lack of oxygen and poor return circulation. The Dunlop vapor furnishes oxygen to these congested parts, promotes normal cell activity and thereby growth. The hemoglobin of red cells absorbs oxygen wherever it can get it. The vapor immediately restores the bright red color which oxygen gives to blood and stimulates circulation, thus supplying food upon which the newly formed cells exist. By pressure, it forces microorganisms and their toxins out into the healthy tissue, where the leucocytes have an opportunity to combat them, and wherever it carries these microorganisms, it also places the borated oxygen vapor, which produces active hyperemia, thereby adding to the vitality of the parts. This has the effect of autogenous vaccine, which produces antibodies. These antibodies add vital resistance to the system by raising the opsonic index against the infecting organisms.

Pickerill criticizes the polishing of tooth surfaces, for fear of mechanically removing Nasmyth's membrane, through which, after the eruption of teeth, he claims the outer layer of enamel is being constantly calcified.

The Nasmyth membrane theory I believe to be a fallacy, because this membrane from which the enamel is formed wears off within a few months after the eruption of the tooth. There is very little question, when the teeth are placed in a proper environment and oral prophylaxis is maintained, but that the outer surface of the enamel becomes much harder.

Some time ago I had the privilege of going through Dr. Bunting's laboratory at the University of Michigan, where he is carrying on a great deal of experimental work. As yet, he is not ready to say how it takes place, but he is satisfied that the teeth which have been given monthly prophylaxis treatments for a year or more have a very much harder surface than teeth which have been ground to that same polished surface at one sitting. He has arrived at this conclusion by comparing the depths to which silver nitrate penetrates the enamel of the two. The theory is advanced by some that this is accomplished through the condensation or compression of the enamel rods from the mechanical rubbing of the outer surface of the teeth. To me this seems hardly feasible. According to Pickerill, Bunting, and other investigators, it is probable that lime salts are deposited in the outer surfaces of the enamel.

The teeth are constantly bathed in saliva, and when this is sufficiently charged with calcium salts, it is supposedly depositing these salts in the outer surface of the enamel. When a tooth surface is kept free from bacterial plaques, one of the functions of the saliva is to preserve, if not to increase its calcium content, but when the surface is covered with bacterial plaques, not only is the saliva kept away from it, but acid fermentation is taking place on that surface all the time, and the acid so formed is constantly drawing upon the calcium salts in the enamel to neutralize itself.

Prophylaxis is saving teeth today and is improving general systemic conditions, and I hope that every practitioner here will be stimulated to put as many of his patients as possible under the oral prophylaxis regime, for every dental practitioner should be a specialist in oral prophylaxis and pyorrhea work. If he is watching for and treating pathological conditions of the oral cavity, he can not help but study the conditions which cause its diseases, and if he learns to recognize and remove the cause of dental troubles, he is practicing prophylaxis. I do not believe that we need more pyorrhea specialists, but we need more general practitioners who recognize conditions, which, if not corrected, will produce pathological results. I believe that the pyorrhea specialist who does nothing but pyorrhea work and sends the patient back to the dentist, who in the first place was unable to prevent the development of the disease, will not be a permanent fixture nor a success in the dental profession, because a man who is not treating these conditions does not realize the causes which produce them. A great deal of the dentistry allowed to pass in our dental schools is responsible for many of the pyorrhetic conditions we meet.

I hope we shall all live to see our great institutions of dental education stimulating in the student body as much, or more interest in oral prophylaxis than they now give to prosthetic dentistry.—*Western Dental Journal.*

